# MicroRNA390 Is Involved in Cadmium Tolerance and Accumulation in Rice

**DOI:** 10.3389/fpls.2016.00235

**Published:** 2016-03-01

**Authors:** Yanfei Ding, Yaoyao Ye, Zhihua Jiang, Yi Wang, Cheng Zhu

**Affiliations:** Key Laboratory of Marine Food Quality and Hazard Controlling Technology of Zhejiang Province, College of Life Sciences, China Jiliang UniversityHangzhou, China

**Keywords:** cadmium, microRNA, miR390, *OsSRK*, rice

## Abstract

Cadmium (Cd) is a non-essential heavy metal that is toxic to plants. microRNAs (miRNAs) are 21-nucleotide RNAs that are ubiquitous regulators of gene expression at the post-transcriptional level. Several plant miRNAs, such as miR390, have vital roles in plant growth, development and responses to environmental stresses including heavy metal stress. In this study, the expression of mature miR390 was significantly down-regulated under Cd stress in rice. Consequently, the target gene of miR390, *OsSRK* was dramatically induced by Cd treatment. Transgenic rice plants overexpressing miR390 displayed reduced Cd tolerance and higher Cd accumulation compared with wild-type plants. Simultaneously, expression of *OsSRK* was less pronounced in *35S:MIR390* plants than in wild-type. These results indicate that miR390 was a negative regulator involved in Cd stress tolerance in rice.

## Introduction

Cadmium is an important environmental pollutant that is highly toxic to plants (Schützendübel et al., 2001). It is widely released into soil, air, and water mainly by eﬄuent from industrial sources, mining, and phosphate fertilization (Pinto et al., 2004). Cd is readily taken up by plants, resulting in toxic symptoms. It damages the photosynthetic apparatus, decreases carbon assimilation and chlorophyll content, and eventually leads to wilting and plant death (Hsu and Kao, 2003; Rodriguez-Serrano et al., 2009). Plants have evolved various molecular mechanisms to respond to heavy metal stress, one of which involves microRNA-guided gene regulation at the post-transcriptional level (Huang et al., 2008, 2010; Zhou et al., 2008, 2012). miRNAs are a class of endogenous non-coding small RNAs that can base pair their target mRNAs to induce their degradation or repress their translation in eukaryotic organisms (Bartel, 2004). In plants, increasing reports have demonstrated that miRNAs have vital regulatory roles in growth, development and plant resistance to abiotic and biotic stresses, including heavy metal stress (Sunkar et al., 2006; Ding and Zhu, 2009; Khraiwesh et al., 2012).

microRNA390 (miR390) is conserved in rice (*Oryza sativa*), maize (*Zea mays*), and *Arabidopsis thaliana*. In rice, it is represented by one member with one locus, whereas in *Arabidopsis* and poplar (*Populus trichocarpa*), it is represented by two members with three and six loci, respectively (Sunkar et al., 2005). In *Arabidopsis*, miR390 exerts its action through the biogenesis of ta-siRNAs that, in turn, leads to the degradation of *ARFs* (auxin response factors) that play critical roles in lateral root development (Marin et al., 2010; Meng et al., 2010). Recent studies indicate that it also plays an important role in plant stress tolerance because it was up-regulated under drought stress in cowpea (*Vigna unguiculata*; Barrera-Figueroa et al., 2011) and *Brachypodium distachyon* (Budak and Akpinar, 2011), while down-regulated under aluminum (Al) treatment in *Medicago truncatula* (Chen et al., 2012). The rice genome encodes miR390, which regulates the expression of *O. sativa stress-responsive leucine-rich repeat receptor-like kinase* (*OsSRK*; LOC_Os02g10100). Cleavage of *OsSRK* mRNA by miR390 has been confirmed by 5′ rapid amplification of cDNA ends (5′ RACE; Sunkar et al., 2005). RLKs belong to the large RLK/Pelle gene family, and are involved in a range of processes, including regulation of development, disease resistance, and biotic and/or abiotic stress responses (Osakabe et al., 2005; Ouyang et al., 2010). However, the role of miR390 in the rice stress response and the biological consequences of target gene regulation of miR390 remain to be identified.

In this study, a transgenic approach was used to investigate the role of miR390 in the Cd stress response in rice. Whereas, mature miR390 was found to be down-regulated by Cd stress in rice, *OsSRK* transcripts were dramatically induced under Cd stress. Overexpression of miR390 significantly retarded seedling growth under Cd stress conditions compared with wild-type plants. MiR390-overexpressing plants showed higher accumulation of Cd and a higher degree of Cd-induced oxidative stress than wild-type. These results provided a link between miR390 and Cd stress tolerance in rice.

## Materials and Methods

### Plant Treatment and RNA Isolation

Seeds of rice Zhonghua 11 (*O. sativa* L. subsp. *japonica*) were germinated in an incubator at 37°C in the dark. Uniformly germinated seeds were then transferred to a pot under a 13 h light (29°C)/11 h dark (22°C) photoperiod. For Cd treatment, 2-weeks-old seedlings were transferred to plastic containers containing Yoshida nutrient solution with 60 μM CdCl_2_, and the roots were harvested at 1, 3, 6, 12, 36, and 48 h post-treatment. Untreated seedlings were used as controls. The total RNA from roots was extracted using the Trizol reagent (Invitrogen).

### Real-Time Quantitative PCR Analysis

Total RNA extracted from rice roots after exposure to 60 μM CdCl_2_ for 0, 1, 3, 6, 12, 36, and 48 h was separately isolated using Trizol reagent (Invitrogen) and treated with 5 U of RNase-free DNase I (TaKaRa) to remove DNA contamination from the RNA. The differentially expressed mature miR390 were validated by stem–loop RT-PCR as previously reported (Chen et al., 2005; Varkonyi-Gasic et al., 2007). Real-time PCRs were carried out using a Rotor-Gene Q machine (parameters: 95°C for 1 min, followed by 45 cycles of 95°C for 10 s, 58°C for 15 s, and 72°C for 15 s). Experiments were performed in triplicate and the results were represented by mean ± SE of three replicates. Normalized expression levels were calculated with the 2^-ΔΔC(t)^ method using U6 as the internal reference gene. Primers used in stem–loop RT-PCR and real-time PCR are listed as follows: miR390 RT primer, 5′-GTCGTATCCAGTGCAGGGTCCGAGGTATTCGCACTGGATACGACGGCGTA-3′; forward, 5′-GCCGAAGCTCAGGAGG-3′; reverse, 5′-GTGCAGGGTCCGAGGT-3′. U6 RT primer, 5′-ATTTGGACCATTTCTCGATTTGT-3′; forward, 5′-CGATAAAATTGGAACGATACAGA-3′; reverse, 5′-ATTTGGACCATTTCATTTGT-3′. *OsACTB* forward, 5′-GCCGTCCTCTCTCTGTATGC-3′; reverse, 5′-GGGGACAGTGTGGCTGAC-3′. *OsSRK* (LOC_Os02g10100) forward, 5′-CCTTCGCAAACTTCTCCG-3′; reverse, 5′-ACTGCCTCCTTCTCAACA-3′.

### Sequence Alignment and *cis*-acting Element Analysis of miR390 and its Target

The pre-miR390 sequence was downloaded from miRBase, miRBase Release 18.0^1^. The 1500-bp DNA sequences upstream of the transcription initiation site of the pre-miR390 gene and *OsSRK* were extracted from the TIGR Rice Genome Annotation site^2^. These sequences were then checked by using PlantCARE^3^ (Lescot et al., 2001).

### Generation and Identification of Transgenic Rice Overexpressing miR390

The fragment of miR390 precursor was amplified from genomic DNA with the following primer pairs: forward, 5′-CGGGGTACCGGAGAGATGTTTTGAGGAAGG-3′; reverse, 5′-AACTGCAGCAGATTTAATTGGTCGTGTGG-3′. The amplified fragments were introduced into p1301-35S-NOS between 35S promoter and NOS terminator with *Kpn*I and *Pst*I enzymes. Cultivars of *japonica* rice Zhonghua 11 were used for transformation. Transgenic lines were achieved by co-cultivation of rice calli with *Agrobacterium tumefaciens* strain EHA105 containing p1301-35S:MIR390 (Hiei et al., 1994). All the putative T0 transgenic plants were screened using PCR analysis with genomic DNA from their leaves. The PCR was performed with primers hpt-F and hpt-R in a 20-μL reaction system. Transgenic rice seedlings were selected using 50 mg/L hyg. Expression levels of mature miR390, *OsSRK*, and metal transporter genes in transgenic rice plants were examined using real-time PCR. *OsHMA2* forward, 5′-TGGCACCACAAAAGGCTATT-3′; reverse, 5′-CACCGTCGATTGGAATGACT-3′; *OsNRAMP5* forward, 5′-AGTGGTTACAGGGAGGCATC-3′, reverse, 5′-GTCTTCCTCGATAGCACCAAG-3′. Quantification of gene expression was done using the comparative CT method. Experiments were performed in triplicate and the results were represented by mean ± SE of three replicates. GUS staining was also used to confirm the transgenic plants (Jefferson, 1987). Histochemical assay of GUS staining was performed by incubating the transgenic plants in reaction mixture comprising: 50 mM phosphate buffer (pH: 7.0) containing 1 mM 5-bromo-4-chloro-3-indolyl glucuronide (X-gluc), 5% methanol, 10 μg/mL cycloheximide, and 1 mM dithiothreitol. The reaction was done overnight at 37°C, followed by clearing with 70% ethanol.

### Effect of Cd Stress on Growth of Rice Seedlings

For the phenotypic analysis of transgenic rice, T1 transgenic rice plants were selected using 50 mg/L hyg. Three weeks after germination, transgenic and wild-type rice seedlings showing consistent growth were transferred to 100 and 200 μM CdCl_2_ Yoshida nutrient solution. The phenotypes of the plants were examined and photographed after Cd treatment.

For physiological and biochemical analysis of transgenic rice, T1 transgenic rice seedlings were selected using 50 mg/L hyg and then cultured with Yoshida’s culture solution for 6 weeks. After exposure to 150 μM CdCl_2_ for 14 days, the chlorophyll, malonyldialdehyde (MDA), and hydrogen peroxide (H_2_O_2_) content was determined. All experiments were performed in triplicate. Data points were presented as the mean ± standard deviation (SD) of three replications. Significant differences among wild-type and transgenic lines were analyzed using the Student’s *t*-test.

The chlorophyll content was measured as described by Knudson et al. (1977). About 100 mg of rice leaves excised to 2–3 cm in length were immersed in 2 ml 95% ethanol for 36 h in the dark. The absorbance of the extracts was read at 645 and 663 nm. The total chlorophyll content was then calculated.

The level of lipid peroxidation in the rice leaves was determined in terms of the peroxidation byproduct MDA following the method of Wang et al. (2009). About 0.1 g of plant leaves was homogenized in 1 ml of 10% trichloroacetic acid (TCA). After the homogenate was centrifuged at 12,000 × *g* for 10 min at 4°C, 2 ml of the supernatant was added 2 ml of 0.6% thiobarbituric acid (TBA) solution. The mixtures were heated at 95°C for 30 min and then cooled quickly on ice. The resulting mixtures were centrifuged at 10,000 × *g* for 10 min and the absorbance of the supernatants was measured at 450, 532, and 600 nm.

The H_2_O_2_ content was determined following the method of Jana and Choudhuri (1982). About 0.1 g of leaf segments were ground into powder and homogenized in 3 mL of pre-cooled 50 mM phosphate buffer (pH 6.5). After centrifugation at 6000 × *g* for 25 min, 1 mL of 0.1% titanium sulfate in 20% (v/v) H_2_SO_4_ was added to 3 mL of the resultant suspension. The mixture was immediately centrifuged at 6000 × *g* for 15 min. Absorbance of the supernatant was recorded at 410 nm. The content of H_2_O_2_ was confirmed using the extinction coefficient of 0.28 μM^-1^ cm^-1^, and expressed as μmol g^-1^ FW.

### Cd Content Determination

For determination of Cd, roots of rice seedlings after 150 μM CdCl_2_ treatment for 14 days were immersed in 20 mM disodium ethylenediamine tetra-acetic acid (Na_2_-EDTA) for 20 min and then rinsed three times with deionized water (He et al., 2008). The roots, stems (containing the leaf sheath) and leaves were dried at 105°C for 2 h and then at 70°C to achieve a constant weight. 0.1 g DW of plant material was digested with a mixture of HNO_3_: HF (6:2, v/v) at 120°C with a Multiwave until they were completely digested. Cd was then quantified using an atomic absorption spectrometer (AA-7000, Shimadzu, Tokyo, Japan). The amount of Cd was expressed on the basis of DW.

## Results

### Cd Stress Decreases miR390 Level

**FIGURE 1:**
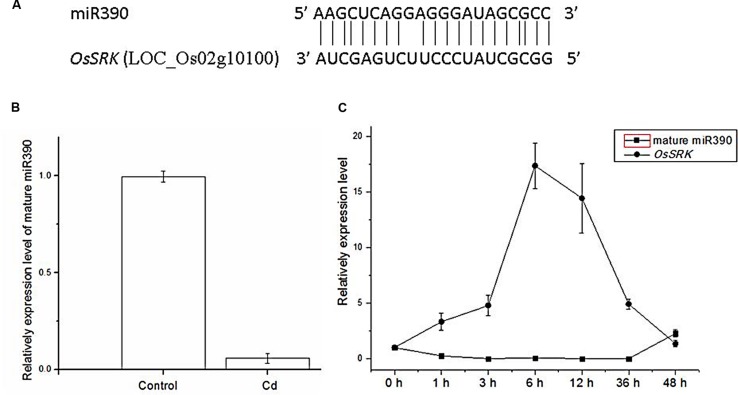
**Expression levels of miR390 and its target gene *OsSRK* as detected by qPCR. (A)** Sequence alignment of miR390 and *OsSRK*. The matched base pairs are indicated by a vertical line. **(B)** Detection of the transcript of mature miR390 expression from 2-weeks-old rice seedlings treated with 60 μM CdCl_2_ for 6 h by qPCR. *U6* gene was used as the inner control. **(C)** QPCR analysis of the mature miR390 and *OsSRK* transcript levels in roots of 2-weeks-old rice seedlings treated for the indicated times with 60 μM CdCl_2_. Quantifications were normalized to the expression of *OsACTB*. Error bars represent the SE for three independent experiments.

Rice miRNA expression patterns were examined under Cd stress using a microarray assay containing probes complementary to the miRNA sequences in miRBase Release 11.0^4^ (Ding et al., 2011). 1-week-old rice seedlings were treated with 60 μM CdCl_2_ for 6 h, after which small RNA was isolated from roots and used to screen the microarray. Microarray results showed that miR390 expression was substantially down-regulated under Cd stress (Ding et al., 2011). In this study, we performed real time PCR analysis of the expression of mature miR390 in 2-weeks-old rice seedlings. Mature miR390 expression was down-regulated after 6 h exposure to 60 μM CdCl_2_, which was consistent with the microarray data (**Figure 1B**).

To examine time-dependent changes in the expression of mature miR390, qPCR analysis was performed in 2-weeks-old rice seedlings. In rice roots, miR390 expression decreased after 1 h of treatment with 60 μM Cd, and continued to decrease over 36 h of the same treatment (**Figure 1C**). By contrast, the target gene of miR390, *OsSRK*, was induced sharply after exposure to Cd for 36 h (**Figure 1C**). These results showed that the profiles of miR390 and *OsSRK* transcripts were complementary, but not exactly opposite, to each other.

### Sequence Analysis of miR390 and *OsSRK*

MicroRNA sequences were derived from miRBase^5^. Sequence alignment of miR390 and *OsSRK* was as shown in **Figure 1A**. Upstream *cis*-acting regulatory element sequences of miR390 and *OsSRK* were analyzed by plantCARE^6^. Stress- and defense-related elements, such as ARE, TC-rich repeats and MBS, were enriched in these regions, suggesting that miR390 and its target *OsSRK* were possibly involved in the stress responses in plants (**Table 1**).

**Table 1:** Stress-related *cis*-elements analysis of miR390 and its target *OsSRK.*

	Site name	Loc (-bp)	
miR390	ARE	-1146	
	LTR	-1341	-900
	MBS	-737	-881
	TC-rich repeats	-234	
	TGACG-motif	-914	
*OsSRK*	ARE	-1141	-522
	MBS	-179	
	TC-rich repeats	-390	
	TCA-element	-85	
	TGA-element	-607	

*ARE, anaerobic responsive element; LTR, low temperature-responsive element; TC-rich repeats, defense and stress responsive element; TGA-element, auxin responsive element; TGACG-motif, MeJA-responsive element; TCA-element, salicylic acid-responsive element; MBS, MYB binding site, involved in drought-inducibility*.

### 35S:MIR390 Plants are More Sensitive to Cd Stress than are Wild-Type Plants

**FIGURE 2:**
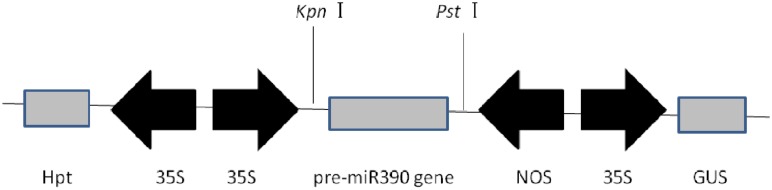
**Expression in plants of the vector of p1301-*35S:MIR390***. 35S, CaMV 35S promoter; NOS, nopaline synthase terminator; *GUS*, gene encoding β-glucuronidase; *Hpt*, gene encoding hygromycin phosphotransferase.

**FIGURE 3:**
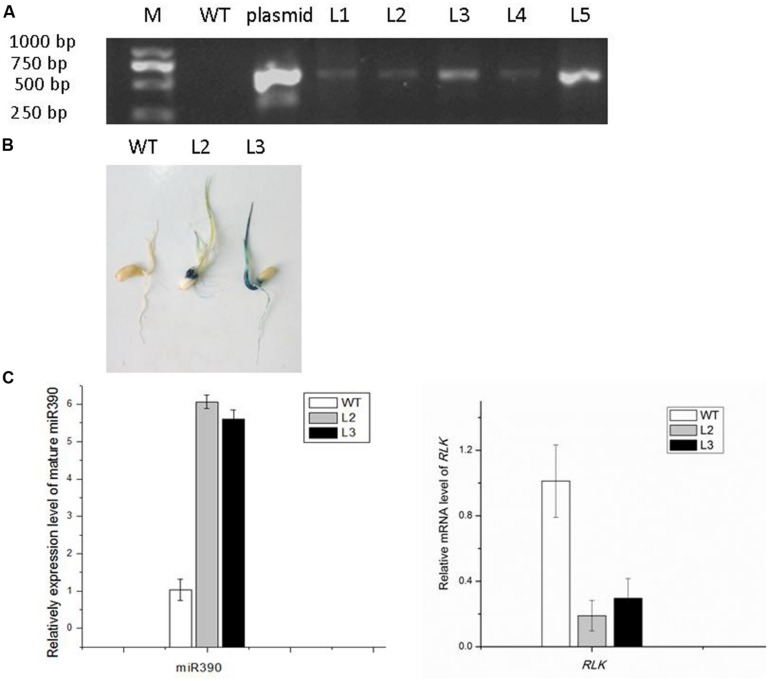
**Confirmation of *35S:MIR390* transgenic rice plants. (A)** Genomic PCR analysis of transgenic rice plants. Genomic DNA was used as the template for PCR. The expected size of the product was 621 bp. Key: M, DNA marker; WT, wild-type plant; L1–L5, transgenic plants. **(B)** GUS activity analysis of transgenic rice plants. **(C)** qPCR analysis of the mature miR390 and *OsSRK* levels in wild-type and transgenic rice.

To determine whether miR390 influences plant growth under heavy metal conditions, transgenic rice plants overexpressing miR390 under control of the CaMV 35S promoter (*35S:MIR390* plants) were generated. The hygromycin (hyg) phosphotransferase gene (*hpt*) was inserted in T-DNA as a selectable marker (**Figure 2**). The transgenic *35S:MIR390* rice plants were single-locus T-DNA insertion lines. T0 transgenic plants were verified by qPCR and GUS activity assay (**Figure 3**). Transcript levels of the mature miR390 were substantially increased in transgenic plants, indicating that *35S:MIR390* plants overexpress miR390. *OsSRK* transcript levels were lower in the transgenic plants than in wild-type controls. These results further validated the negative regulation of miR390 in *OsSRK* transcripts.

**FIGURE 4:**
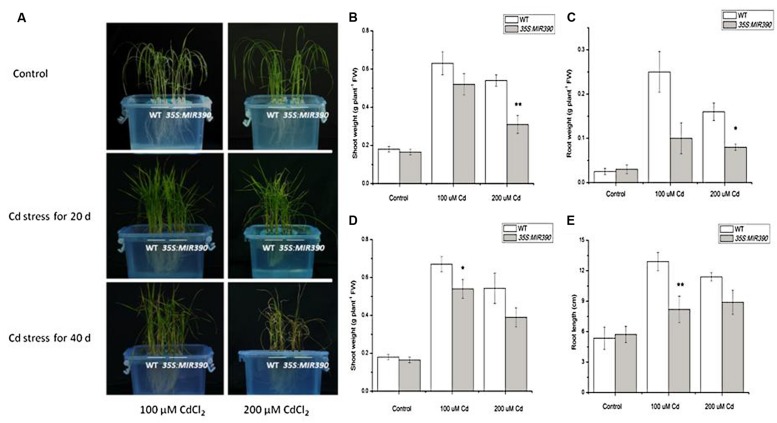
**Decreased Cd resistance of *35S:MIR390* transgenic rice plants. (A)** Phenotypic comparison of wild-type and *35S:MIR390* transgenic rice plants under Cd stress. In each growth chamber, WT plants were grown on the left and *35S:MIR390* plants on the right. Upper panel: control, plants grown for 3 weeks under normal conditions before treatment. Middle panel: 3-weeks-old plants treated with 100 μM CdCl_2_ (left) and 200 μM CdCl_2_ (right) for 20 days. Lower panel: 3-weeks-old plants treated with 100 μM CdCl_2_ (left) and 200 μM CdCl_2_ (right) for 40 days. **(B,C)** Shoot fresh weight (FW) and root FW of wild-type and *35S:MIR390* transgenic plants grown under Cd stress for 20 days. **(D,E)** Shoot FW and root length of wild-type and *35S:MIR390* transgenic plants grown under Cd stress for 40 days. In **(B–E)**, significant differences from the wild-type as determined by the Student’s *t*-test are indicated (^∗^*P* < 0.05, ^∗∗^*P* < 0.01). Bars indicate SE.

**FIGURE 5:**
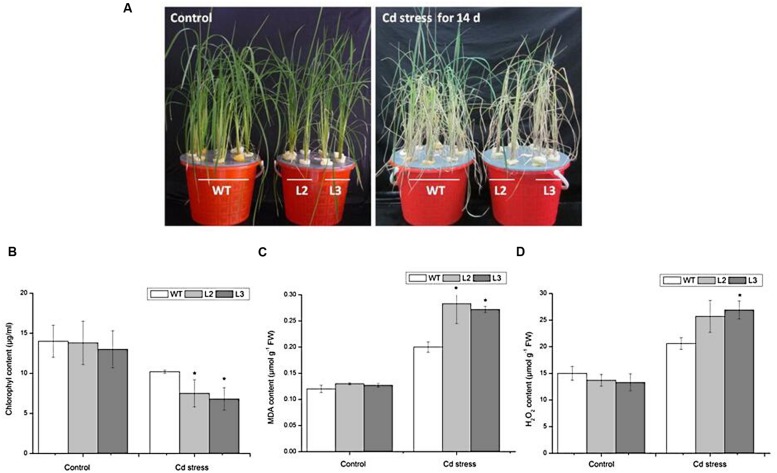
**Performance of wild-type and *35S:MIR390* transgenic plants treated with 150 μM CdCl_2_ for 14 days. (A)** Phenotypic comparison of WT and 35S:MIR390 transgenic rice. **(B)** Chlorophyll content. **(C)** MDA content. **(D)** H_2_O_2_ content. Each column represents an average of three replicates, and bars indicate SE. ^∗^Indicate significant differences from between wild-type and transgenic plants at *P* < 0.05.

We examined the growth of miR390-overexpressing rice seedlings under Cd stressed conditions (**Figures 4A** and **5A**). When the plants were grown under normal conditions, no noticeable differences in seedling growth were observed between wild-type and transgenic plants. However, the plant growth of transgenic lines was dramatically reduced compared with that of wild-type plants under Cd stress (**Figure 4A**). Quantitative analyses confirmed that the shoot weight, lateral root weight and root length of *35S:MIR390* plants were significantly lower than those of wild-type plants when grown on Cd (**Figures 4B–E**). The chlorophyll content was also determined. Under normal conditions, no significant differences in chlorophyll content were observed among the plants. However, *35S:MIR390* lines had less chlorophyll under Cd stress compared with wild-type controls (**Figure 5B**).

Malonyldialdehyde is an indicator of oxidative attack on membrane lipids. When subjected to Cd stress, MDA levels increased in wild-type rice seedlings, which attributed to the Cd-induced oxidative stress, in accordance with previous experiments (Shah et al., 2001; Guo et al., 2004). However, in Cd-stressed *35S:MIR390* lines, MDA content increased by 40% compared with wild-type plants (**Figure 5C**). These results indicated that overexpression of miR390 can increase membrane damage caused by Cd stress.

Stresses usually cause damage in plants via oxidative stress involving the generation of reactive oxygen species (ROS), such as H_2_O_2_ (Apel and Hirt, 2004). Therefore, the H_2_O_2_ content in leaves of wild-type and transgenic plants were examined. As shown in **Figure 5D**, there were no significant differences in H_2_O_2_ content under normal conditions. After 14 days of 150 μM CdCl_2_ treatment, the H_2_O_2_ content in leaves of wild-type increased by 1.4-fold, whereas those in transgenic plants increased by 2.0-fold, suggesting that overexpression of miR390 cannot efficiently eliminate H_2_O_2_ produced under Cd stress, resulting in the decreased Cd tolerance of transgenic rice. All these results indicated that the *35S:MIR390* plants were Cd sensitive.

### 35S:MIR390 Plants Accumulate more Cd than Wild-Type Plants

**FIGURE 6:**
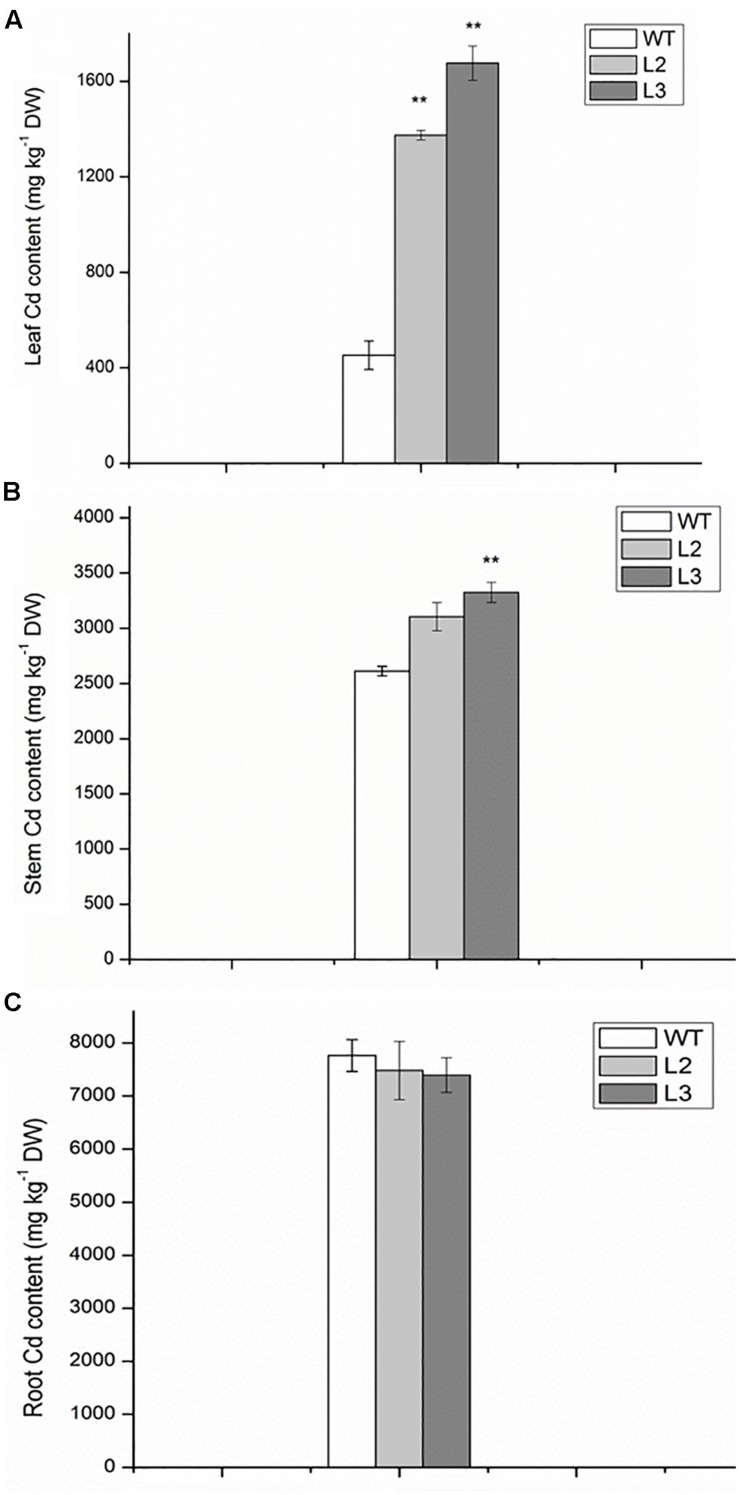
**The contents of Cd in leaves **(A)**, stems (containing leaf sheaths) **(B)** and roots **(C)** of wild-type and transgenic plants exposed to 150 mM CdCl_2_ for 14 days**. Data are presented as the mean ± SD; ^∗∗^ indicates significant differences at *P* < 0.01 between wild-type and transgenic plants.

To assess whether the increase in Cd sensitivity was because of higher metal accumulation, the Cd content in roots and shoots of miR390-overexpressing rice lines was compared with that of the same organs of wild-type plants. The differences in Cd concentration of roots were not significant between wild-type and transgenic lines, whereas the Cd levels were higher in the leaves and stems of transgenic lines than in wild-type plants (**Figure 6**). Thus, compared with wild-type plants, *35S:MIR390* plants enhanced Cd accumulation, which might result in the sensitivity of *35S:MIR390* plants to Cd stress.

**FIGURE 7:**
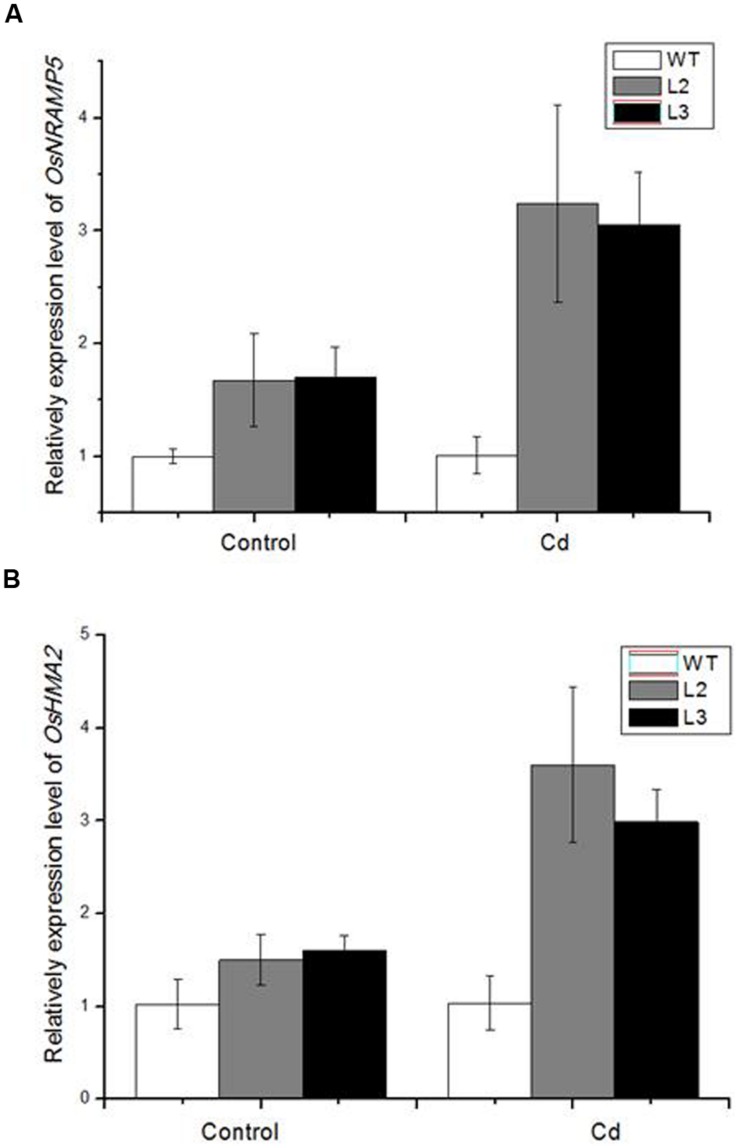
**Quantitative real-time PCR (QPCR) analysis of metal transporter genes in *35S:MIR390* transgenic plant roots compare with wild-type plants under Cd stress. (A)** *OsNRAMP5*. **(B)** *OsHMA2*. Normalized expression levels were calculated with the 2^-ΔΔC(t)^ method using *OsACTB* as the internal reference gene.

To estimate the effect of miR390 on Cd accumulation and translocation to shoots, several heavy metal transporter genes were examined in *35S:MIR390* roots after Cd treatment by qPCR (**Figure 7**). In our analyses, more transcripts of *OsHMA2* and *OsNRAMP5* were produced in the Cd-stressed roots of *35S:MIR390* than in wild-type plants, indicating that they were positively regulated by miR390 and important for Cd accumulation and translocation in rice.

## Discussion

Characterization of the role of miRNAs in the plant stress response is an active field of research. Recently, several methods have been used for the dissection and characterization of diverse miRNAs involved in plant responses and adaptations to heavy metal stress (Sunkar and Zhu, 2004; Zhou et al., 2008; Chen et al., 2012). For example, miRNA microarray assays led to the identification of 19 Cd-responsive miRNAs in rice (Ding et al., 2011). Deep sequencing studies revealed that 52 new miRNA candidates were differentially regulated by mercury Hg(II) in *M. truncatula* (Zhou et al., 2012). Although, an increasing number of heavy metal-related miRNAs has been identified, their precise roles remain to be verified. Overexpression of miRNAs and/or miRNA targets or knockout mutants of target genes have proved useful for analyzing the function of stress-responsive miRNAs and further uncovering miRNA-regulated transcripts upon application of stress. Transgenic strategies have also been used to improve the heavy metal stress tolerance of plants. Zhang et al. (2013) reported that miR395 overexpressing rapeseed (*Brassica napus*) showed a lower degree of Cd-induced oxidative stress than wild-type plants, suggesting that miR395 would be involved in detoxification of Cd in *B. napus*. This study was the first to report that overexpression of a miRNA can affect heavy metal tolerance of rice.

Previous studies suggested that the miR390 family was involved in the plant heavy metal stress response (Mendoza-Soto et al., 2012; Yang and Chen, 2013). miR390 has been reported to be down-regulated by Al, As, Cd, and Hg (Ding et al., 2011; Chen et al., 2012; Zhou et al., 2012). In this study, mature miR390 was decreased in roots of rice under Cd stress, which was consistent to the expression pattern of miR390 precursor in our previous study (Ding et al., 2011). The transgenic plants overexpressing miR390 showed enhanced sensitivity to Cd stress. MiR390-overexpressing plants had higher accumulation of Cd and higher translocation of Cd from roots to shoots relative to the wild-type. Furthermore, expression of miR390 changed the expression pattern of several metal transporter genes. Results showed that *OsHMA2* and *OsNRAMP5* were increased in miR390-overexpressing transgenic plants compared to wild-type plants under Cd treatment. *OsHMA2* was reported to be a major Zn/Cd transporter from roots to shoots (Takahashi et al., 2012). In this study, the expression level of *OsHMA2* was increased in *35S:MIR390* plants, in which Cd contents were higher in the shoots compared with wild-type plants. Thus, the increase in Cd translocation of *35S:MIR390* plants could be attributable to the change in the expression level of *OsHMA2*. In addition, *OsNRAMP5*, was reported to be a Mn and Cd transporter involved in the root uptake of these metals from the medium (Ishimaru et al., 2012). More *OsNRAMP5* transcripts were detected in *35S:MIR390* roots, implying more Cd accumulation in rice.

Plant miRNAs execute biological functions via negative regulation of their specific target genes. In *Arabidopsis*, miR390 was demonstrated to mediate the regulatory pathway miR390-TAS3-ARFs involved in auxin signaling and the regulation of lateral root development (Marin et al., 2010; Yoon et al., 2010). In this study, we investigated lateral root growth in *35S:MIR390* transgenic lines in response to Cd stress. Quantitative analyses showed that lateral root weight and root length of *35S:MIR390* plants were significantly lower than those of wild-type plants when grown on Cd. However, no reduction in lateral root number was observed in *35S:MIR390* seedlings treated with Cd. These results were partly consistent with Yoon et al.’s (2010) report in which miR390 exerted a negative effect on lateral root formation in *Arabidopsis*. The role of miR390-TAS3-*ARFs* pathway on lateral root development in rice remains to be validated and elucidated.

In rice, miR390 has been experimentally validated to target a *stress-responsive LRR-like kinase* (*OsSRK*; LOC_Os02g10100) by 5′ RACE and degradome sequencing (Sunkar et al., 2005; Zhou et al., 2010). RLKs are transmembrane proteins containing an extracellular LRR and a Ser/Thr kinase domain, and can activate a complex array of intracellular signaling pathways in response to various plant environmental and developmental signals (Tichtinsky et al., 2003; Osakabe et al., 2005; Chae et al., 2009). *RLKs* include multigene families. To date, at least 1131 *RLK* genes have been identified in rice, several of which have been reported to play important roles in plant stress response (Shiu et al., 2004). Transgenic rice plants with overexpression of a putative *RLK* gene, *OsSIK1* showed higher tolerance to salt and drought stresses than wild-type plants (Ouyang et al., 2010). In germinating rice seeds exposed to excess copper (Cu), a *RLK* protein spot was identified to be up-regulated by MALDI-TOF mass spectrometry (Ahsan et al., 2007). A receptor-like protein gene, *OsRMC* was reported to be involved in regulation of Fe acquisition in rice (Yang et al., 2013). In this study, the expression of *OsSRK* (Os02g10100) was up-regulated after exposure to Cd stress in rice seedlings. *OsSRK* was repressed in *35S:MIR390* plants, in which the expression levels of several metal transporter genes changed. However, the relationship between *OsSRK* and metal transporter genes needs further research. Gain- and loss-of-function plants targeting *OsSRK* would be helpful to determine the role of *OsSRK* in regulation of plant response to heavy metal stress.

## Author Contributions

YD and CZ conceived and designed experiment; YY, ZJ, and YW carried out experiments; YD, YY, ZJ, and YW analyzed experimental results; YD and YY wrote the manuscript; All authors read and approved the manuscript.

## Conflict of Interest Statement

The authors declare that the research was conducted in the absence of any commercial or financial relationships that could be construed as a potential conflict of interest.

## Footnotes

1 http://microrna.sanger.ac.uk/

2 http://www.mirbase.org/cgi-bin/mirna_entry.pl?acc=MI0001690

3 http://bioinformatics.psb.ugent.be/webtools/plantcare/html/

4 http://www.tigr.org/tdb/e2k1/osa1/data_download.shtml

5 http://microrna.sanger.ac.uk/sequences/

6 http://bioinformatics.psb.ugent.be/webtools/plantcare/html/

## ABBREVIATIONS

CaMV: cauliflower mosaic virus
Cd: cadmium
miRNA: microRNA
qPCR: quantitative real-time PCR
RLK: receptor-like kinase
RT-PCR: reverse transcription PCR
